# Switching Chemoselectivity: Using Mechanochemistry to Alter Reaction Kinetics

**DOI:** 10.1002/anie.201810141

**Published:** 2018-11-08

**Authors:** Joseph L. Howard, Michael C. Brand, Duncan L. Browne

**Affiliations:** ^1^ School of Chemistry Cardiff University Main Building, Park Place Cardiff CF10 3EQ UK

**Keywords:** ball milling, chemoselectivity, mechanochemistry, reaction kinetics, solventless reactions

## Abstract

A reaction manifold has been discovered in which the chemoselectivity can be altered by switching between neat milling and liquid assisted grinding (LAG) with polar additives. After investigation of the reaction mechanism, it has been established that this switching in reaction pathway is due to the neat mechanochemical conditions exhibiting different kinetics for a key step in the transformation. This proof of concept study demonstrates that mechanochemistry can be used to trap the kinetic product of a reaction. It is envisaged that, if this concept can be successfully applied to other transformations, novel synthetic processes could be discovered and known reaction pathways perturbed or diverted.

Mechanochemistry is emerging as a technique for synthesis with several synthetically important processes now reported in ball milling devices and rapid growth in recent times.^1^, ^2^ Furthermore, it has been shown that, for certain examples, significant advantages over solution chemistry can be obtained, such as a decrease in reaction time or improvement in selectivity.1k There are also examples where the use of mechanochemistry can alter reactivity, resulting in different products when compared to solution or liquid assisted grinding (LAG).1k,1l For example, previous work in our group has shown the use of LAG to control the selectivity of mechanochemical fluorination (Scheme 1 a).^3^ It has also been shown that by using mechanochemical conditions, the position of equilibrium can be altered, such as in disulfide metathesis reactions for example (Scheme 1 b).^4^ This demonstrates that it is possible to alter the thermodynamic product of a reaction by conducting it in the solid‐state under ball milling and in the latter instance, crystal lattice energies help drive the process. The majority of examples are limited to different possible outcomes from the same reaction pathway. However, work by Mack and co‐workers demonstrated the possibility of using LAG to change the reaction pathway (Scheme 1 c).^5^ It was found when performing a palladium catalysed alkyne‐alkyne (Glaser–Hay) coupling that on using a non‐polar LAG additive, the diyne product was obtained but with a polar LAG additive, the enyne was produced. However, the origins of the different reaction outcomes observed in this latter example remain elusive. As it has already been shown that mechanochemical conditions can alter both the thermodynamics^4^ and kinetics^6^ of covalent bond forming reactions, we envisaged exploiting these possibilities to alter the course of a reaction, leading to alternative products (Scheme 1 d). Indeed, we have serendipitously discovered a reaction manifold that exhibits significantly different behavior under neat or non‐polar LAG mechanochemical conditions compared to solution or polar LAG conditions. Initially, difluorinated diketone **1** was milled in the presence of cesium carbonate and phenyl disulfide to afford tetrafluorohydroxyketone **2** which was isolated in 72 % yield (Table 1, entry 1). The unreacted disulfide could also be isolated quantitatively from this reaction mixture. This product/observation is in contrast to the result reported for the same reagents in solution by Yi, Lu, and co‐workers, who report the formation of the difluorothioether compounds **3 a** and **3 b**.^7^ Encouraged by the significantly different reactivity observed under mechanochemical conditions, this reaction was investigated further. It was found that under LAG conditions, using DMSO, the reactivity could be switched completely to thioether products **3 a** and **3 b**. This switch is dependent on the quantity of DMSO (Table 1, entries 2–5). Beyond 50 μL, none of the fluorinated alcohol **2** was observed, and the highest yield of **3 a** was achieved by milling with 50 μL of DMSO. This is therefore an example of a reaction where the pathway can be completely altered by neat milling. Under neat milling conditions, the disulfide is untouched, whereas use of LAG or solution phase reaction conditions leads to the consumption of disulfide and formation of the thioether products **3 a** and **3 b**.

**Scheme 1:**
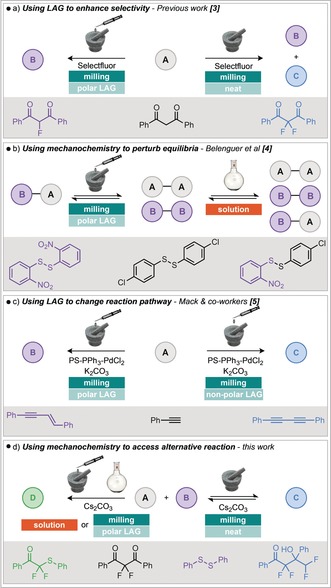
Examples of using mechanochemistry to alter reactivity by different methods.

**Table 1:** Effect of different liquid additives on reaction selectivity. 
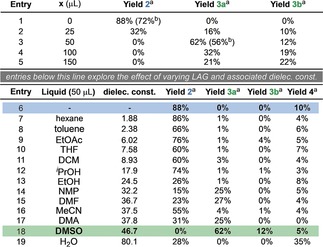

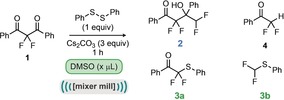

[a] Yield determined by ^19^F NMR spectroscopy with α,α,α‐trifluorotoluene as an internal standard. [b] Yield of isolated product.

Having established that liquid assisted grinding has a significant effect on the outcome of this reaction, it was hypothesized that the nature of the solvent could lead to different results. Indeed, it has been observed previously that solvents of different polarities can be used for LAG to form different polymorphs of cocrystals.^8^ We therefore tested a wide range of solvents, with varying dielectric constants (Table 1, entries 7–19). It can be seen that the reactivity can again be switched, depending on how polar the solvent is. The most polar solvents tested (*ϵ*>30, entries 14–19) seem to favour the reaction with disulfide to form thioether **3 a** (with the exception of acetonitrile and water). Indeed, in the case of water there appears to be very little discrimination between the reaction products with the major component of this reaction being the difluoroketone **4**, thus suggesting that water is not a critical factor in determining the selectivity. However, the less polar solvents (entries 7–13) appear to favour the formation of **2**. These intriguing observations are, to the best of our knowledge, unprecedented. While there are previous examples where the selectivity, rate or products of a reaction have been changed, switching to a different reaction pathway using neat and LAG milling has not been previously reported. It was therefore important to attempt to propose and understand the mechanism of this process. Our proposed mechanism is presented in Scheme 2, and commences with the fragmentation of difluorodiketone **1**, likely initiated by nucleophilic attack onto one of the ketones. This type of fragmentation has been reported previously on similar structures, and may be enhanced by the motif of three adjacent electropositive carbon atoms.^9^ The product of this fragmentation, enolate **5**, can now react via different pathways, depending on the conditions. It can either attack the electrophilic disulfide to enter into the thioether reaction pathway, or can undergo a self‐aldol reaction with the protonated enolate **4** to yield the observed hydroxyketone product (**2**). The reaction with the disulfide, observed under LAG conditions with the more polar solvents, and also in solution, yields difluorinated thioether **3 a**. Under longer reaction times, or at higher temperatures, this can fragment further to difluoromethylthioether **3 b**.^7^ In order to probe the validity of the proposed mechanism, we designed several control experiments to test the various aspects.

**Scheme 2:**
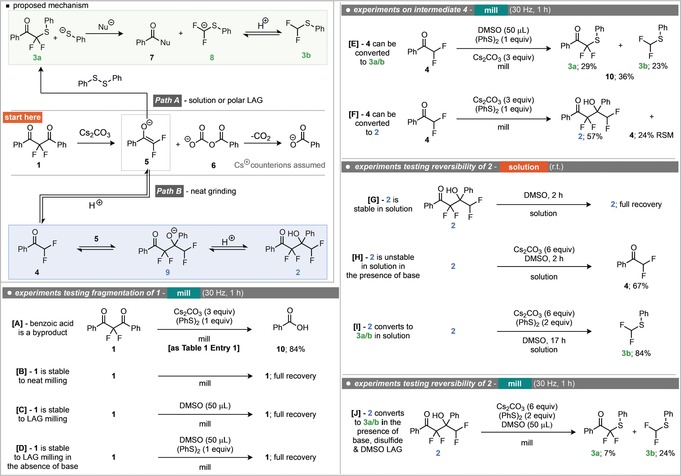
Proposed reaction mechanisms and control experiments. Yield determined by ^19^F NMR compared to α,α,α‐trifluorotoluene as an internal standard.^11^

Initially, the fragmentation of the difluorinated diketone **1** was investigated (Scheme 2, equations A–D). It was observed that under the standard reaction conditions (as shown in Table 1, entry 1), benzoic acid could be isolated as a side product in 84 % yield along with the tetrafluoro alcohol (**2**) (Scheme 2, equation A). This suggests that the identity of the nucleophile initiating the fragmentation could be water or carbonate. However, no water was deliberately added to the reaction, so the only water available would be present in one of the other reagents. Control experiments (Scheme 2, equations B–D) revealed that in the absence of cesium carbonate, no reaction was observed, with only starting material observed in the crude reaction mixtures. The requirement for cesium carbonate supports the notion that CO_3_^2−^ acts as the initiating nucleophile, with subsequent decarboxylation to benzoic acid (as depicted in the proposed mechanism). The next part of the mechanism explored was the presence and identity of the proposed intermediate difluoroketone **4** and its corresponding enolate **5** that is common to both reaction pathways (Scheme 2, equations E and F). Indeed, preparation of ketone **4** and subjection to both neat grinding and LAG conditions yielded the expected products, demonstrating its competence in both reaction pathways and supporting the notion that **4** is an intermediate in both of these processes.^10^ Finally, in order to test whether this process could be under thermodynamic control, the reversibility of each step was examined (Scheme 2, equations G–I). It was found that subjecting tetrafluoro alcohol **2** to stirring in DMSO resulted in no transformation and full recovery of **2** (Scheme 2, equation G). Whereas, stirring alcohol **2** in DMSO with cesium carbonate (no disulfide), led to the generation of ketone **4** in 67 % NMR yield (Scheme 2, equation H). Under analogous conditions, but with inclusion of disulfide, difluoromethylthioether **3 b** was observed in 84 % yield (Scheme 2, equation I). Reversibility was also observed in the mixer mill under LAG conditions. On subjecting alcohol **2** to ball milling for one hour in the presence of phenyl disulfide, cesium carbonate and DMSO, thioethers **3 a** and **3 b** were observed (Scheme 2, equation K). Whilst these experiments support the proposed mechanism, they do not provide an explanation to the origin of the observed chemoselectivity differences. In order to probe this phenomenon further, different reaction times were investigated under both LAG and neat conditions, the results are depicted in Scheme 3. Under extended reaction times in the absence of a liquid additive, thioether **3 a** was observed, in stark contrast to the observed product after 1 hour. However, the formation of **3 a** appears to be slow, with only 27 % observed after 8 hours. It can be seen that initially, self‐aldol product **2** is formed, and is then subsequently serving as a source for difluoro ketone **4**.

**Scheme 3:**
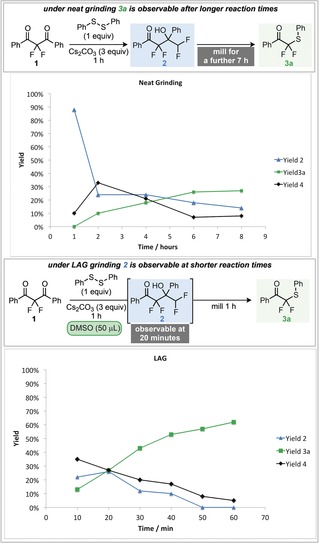
Milling for different times under LAG and neat grinding conditions.

However, upon extended milling durations ketone **4** then slowly reacts with the disulfide to form **3 a**. This suggests that **3 a** is the thermodynamic product of the system. Performing a similar analysis under LAG conditions, demonstrates that at short reaction times (10 minutes), **2** is indeed observable as a kinetic product, also in stark contrast to the initial observations of the system after one hour. Again, **2** is in equilibrium with **4**, which quickly reacts with the disulfide to form **3 a**. This demonstrates that the addition of DMSO has a significant effect on the rate of formation of **3 a** from **4**, with this transformation being significantly faster than under neat milling. The kinetic product of the system is therefore **2**, which is in equilibrium with **4** and can be trapped using mechanochemical conditions without LAG. Under LAG with DMSO, or in solution, the thermodynamic product **3 a** is instead obtained. The physical reasons behind this decrease in reaction rate under mechanochemical conditions is likely due to poor mixing and the consequently non‐homogeneous nature of the reaction mixture. We hypothesise that after the Cs_2_CO_3_ mediated fragmentation of **1**, the surface of any particles of **1** will be coated in enolate **5** (Scheme 4). Upon protonation to ketone **4**, the local concentration of **4** will be much greater than that of the disulfide, so reaction between **4** and **5** to form **2** will be faster than the reaction to generate **3 a**. However, polar solvents will be able to break up this coating of enolate **5**, allowing it to react with the disulfide faster. This hypothesis was tested by subjecting the reaction mixture to different quantities of DMSO under milling for 10 minutes (Scheme 4). It was found that on increasing the quantity of DMSO, the major reaction product switched to **3 a**, demonstrating that higher quantities of DMSO favour reaction with the disulfide to form **3 a**.

**Scheme 4:**
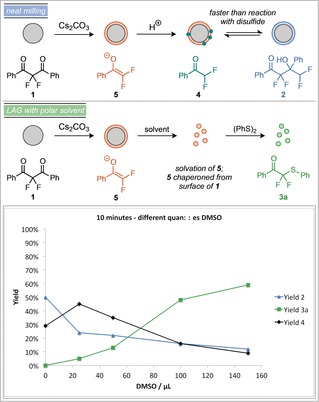
Yields of products after milling for 10 minutes with different quantities of DMSO.^11^

In conclusion, a reaction manifold has been found in which different products are obtained depending on whether it is performed mechanochemically under neat grinding conditions, or under LAG or solution conditions. Polar LAG additives were able to switch the reaction pathway, whereas non‐polar additives were not. After investigating both the mechanism and the behaviour at different reaction times, it has been established that under neat grinding, a kinetic product is being trapped that is not observed under solution or polar LAG conditions. This is the first example of using mechanochemistry to alter the chemoselectivity of a reaction by altering the kinetics, this could lead to screening of other reactions to search for new or overlooked reactivity pathways.

## Conflict of interest

The authors declare no conflict of interest.

## Supporting information

As a service to our authors and readers, this journal provides supporting information supplied by the authors. Such materials are peer reviewed and may be re‐organized for online delivery, but are not copy‐edited or typeset. Technical support issues arising from supporting information (other than missing files) should be addressed to the authors.

Supplementary
