# Supplementary material for: Switching Chemoselectivity: Using Mechanochemistry to Alter Reaction Kinetics
Source: Angew Chem Int Ed Engl. 2018 Nov 8;57(49):16104–8. doi: 10.1002/anie.201810141 (PMC6282732; doi:10.1002/anie.201810141)
Supplement: Supplementary file 1 — Supplementary [file ANIE-57-16104-s001.pdf]

Angewandte Chemie
Eine Zeitschrift der Gesellschaft Deutscher Chemiker

Supporting Information

Switching Chemoselectivity: Using Mechanochemistry to Alter Reaction Kinetics

*Joseph L. Howard, Michael C. Brand, and Duncan L. Browne**

anie_201810141_sm_miscellaneous_information.pdf

General Methods S3

General Procedure for mechanochemical reactions S4

- Liquid Assisted Grinding (LAG) Screen S4
- Optimized conditions for synthesis of (2). General procedure 1 (GP1). S4
- Optimized conditions for synthesis of (3). General Procedure 2 (GP2) S5

Procedures for mechanistic experiments S6

- Testing fragmentation S6
- Testing intermediate S7
- Testing reversibility in solution S8
- Testing reversibility in ball mill S8

Effect of time on yield for LAG reaction S9

Effect of time on yield for neat grinding reaction S10

References S15

Spectroscopic data S16

General Methods

^{1}H, ^{19}F and ^{13}C NMR spectra were obtained on Bruker 400 Ultrashield™ and Bruker 500 MHz spectrometers with chloroform-d as deuterated solvent. The obtained chemical shifts δ are reported in ppm and are referenced to the residual solvent signal. Spin-spin coupling constants *J* are given in Hz.

High resolution mass spectral (HRMS) data were obtained on a Thermo Scientific LTQ Orbitrap XL by the EPSRC UK National Mass Spectrometry Facility at Swansea University or on a Waters MALDI-TOF mx in Cardiff University.

Infrared spectra were recorded on a Shimadzu IR-Affinity-1S FTIR spectrometer. Peaks of medium or higher intensity are reported.

Melting points were measured using a Gallenkamp apparatus and are reported uncorrected.

The ball mill used was an InSolidoTech IST500 mixer mill. Unless otherwise stated, mechanochemical reactions were performed in 14 mL stainless steel jars with one stainless steel ball of mass 4 g at a milling frequency of 30 Hz.

All chemicals were obtained from commercial sources and used without further purification unless stated otherwise.

General Procedure for mechanochemical reactions

Liquid Assisted Grinding (LAG) Screen

2,2-difluoro-1,3-diphenylpropane-1,3-dione (0.065 g, 0.25 mmol.), diphenyl disulfide (0.055 g, 0.25 mmol.), cesium carbonate (0.244 g, 0.75 mmol.) and the liquid additive were added to a 14 mL stainless steel jar and a ball added. The mixture was milled for one hour and the resulting mixture transferred into a flask by manually removing the material with a spatula and washing with ethyl acetate (approximately 40 mL). The insoluble material was removed by gravity filtration. α,α,α-trifluorotoluene (0.020 mL, 0.16 mmol) was added as a standard and ^{19}F NMR was taken of the crude mixture to determine the yields of different products.

Optimized conditions for synthesis of (3a). General procedure 1 (GP1).

2,2-difluoro-1,3-diphenylpropane-1,3-dione (0.065 g, 0.25 mmol.), cesium carbonate (0.244 g, 0.75 mmol), diphenyl disulfide (0.055 g, 0.25 mmol) and DMSO (0.050 mL) were added to a 14 mL stainless steel jar and charged with one stainless steel ball (10 mm, 4.1 g). The reaction mixture was milled at 30 Hz for one hour. The resulting mixture was transferred into a flask by manually removing the material with a spatula and washing with ethyl acetate (approximately 40 mL). The insoluble material was removed by gravity filtration. α,α,α-trifluorotoluene (0.020 mL, 0.16 mmol) was added as a NMR standard and ^{19}F NMR was taken of the crude reaction mixture and the yield determined to be 62%. The mixture was then added to a separating funnel with distilled water (40 mL). The aqueous layer was extracted with ethyl acetate (2 x 40 mL), the organic layers combined, washed with brine (50 mL), dried ($MgSO_4$), and the solvent removed under reduced pressure to yield the crude product. This material was purified by flash column chromatography with gradient elution (0 - 10% ethyl acetate in petroleum ether) to yield the product as a colourless oil (0.038 g, 0.14 mmol., 56%).[1]

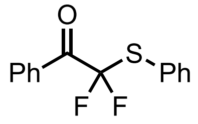

3a

^{1}H NMR (400 MHz, $CDCl_3$) δ 8.13 (d, J = 7.5 Hz, 2H), 7.68 – 7.60 (m, 3H), 7.55 – 7.44 (m, 3H), 7.36 (t, J = 7.3 Hz, 2H).

^{13}C NMR (101 MHz, $CDCl_3$) δ 185.3 (t, *J* = 28.0 Hz), 136.8, 134.8, 131.2, 130.6, 130.5 (t, *J* = 2.5 Hz), 129.4, 128.8, 124.8, 123.8 (t, *J* = 291.0 Hz).

^{19}F NMR (376 MHz, $CDCl_3$) δ -77.20 (s, 2F).

IR 1701, 1597, 1577, 1473, 1448, 1440, 1307, 1269, 1188, 1130, 1056, 1024, 1001, 983, 933, 883, 823, 779, 748, 709, 682, 665, 640, 594, 497, 441, 406 cm^{-1}.

HRMS (EI+): [$C_{14}H_{10}F_2OS$] calc. 264.0420, found 264.0426.

Optimized conditions for synthesis of (2). General Procedure 2 (GP2)

2,2-difluoro-1,3-diphenylpropane-1,3-dione (0.065 g, 0.25 mmol.), cesium carbonate (0.244 g, 0.75 mmol), and diphenyl disulfide (0.055 g, 0.25 mmol) were added to a 14 mL stainless steel jar and charged with one stainless steel ball (10 mm, 4.1 g). The reaction mixture was milled at 30 Hz for one hour. The resulting mixture was transferred into a flask by manually removing the material with a spatula and washing with ethyl acetate (approximately 40 mL). The insoluble material was removed by gravity filtration. α,α,α-trifluorotoluene (0.020 mL, 0.16 mmol) was added as a NMR standard and ^{19}F NMR was taken of the crude reaction mixture and the yield determined to be 88%. The mixture was then added to a separating funnel with distilled water (40 mL). The aqueous layer was extracted with ethyl acetate (2 x 40 mL), the organic layers were combined, washed with brine (50 mL), dried ($MgSO_4$) and the solvent removed under reduced pressure to yield the crude product. This material was purified by flash column chromatography with gradient elution (0 - 10% ethyl acetate in petroleum ether) to yield the product as a yellow oil (0.028 g, 0.09 mmol., 72%).

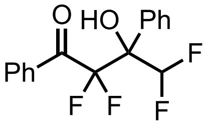

2

^{1}H NMR (400 MHz, $CDCl_3$) δ 7.97 (dd, *J* = 8.5, 1.1 Hz, 2H), 7.81 – 7.57 (m, 3H), 7.55 – 7.37 (m, 5H), 6.43 (t, *J* = 54.5 Hz, 1H), 4.32 (s, 1H). **^{13}C NMR** (101 MHz, $CDCl_3$) δ 190.5 (t, J = 29.5 Hz), 134.9, 132.6 (t, J = 2.5 Hz), 132.6, 130.4 (t, J = 3.5 Hz), 129.4, 128.7, 128.6, 127.0, 115.2 (t, J = 267.0 Hz), 114.0 (tt, J = 250.0, 3.0 Hz), 78.1 (t, J = 23.0 Hz).

^{19}F NMR (376 MHz, $CDCl_3$) δ -105.41 (dt, *J* = 293.5, 8.0 Hz, 1F), -106.63 (dt, *J* = 293.5, 9.3 Hz, 1F), -128.59 (ddt, *J* = 289.3, 54.1, 8.6 Hz, 1F), -130.31 (dddd, *J* = 289.4, 54.9, 10.0, 7.5 Hz, 1F).

IR 3458, 1694, 1597, 1450, 1278, 1161, 1132, 1097, 1070, 846, 808, 744, 711, 553 cm^{-1}.

HRMS (EI+): [$C_{16}H_{12}F_4O_2$] calc. 312.0773, found 312.0772.

Procedures for mechanistic experiments

Testing fragmentation

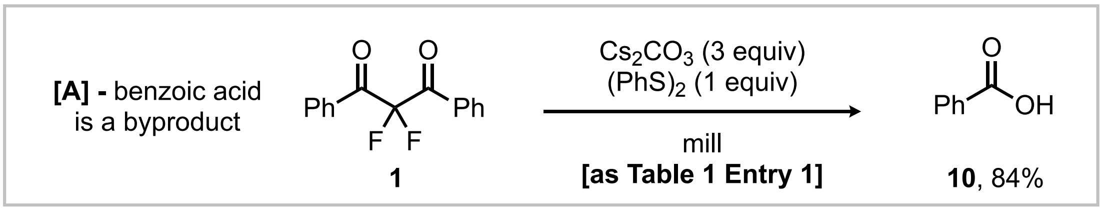

2,2-difluoro-1,3-diphenylpropane-1,3-dione (0.065 g, 0.25 mmol.), cesium carbonate (0.244 g, 0.75 mmol) and diphenyl disulfide (0.055 g, 0.25 mmol) were added to a 14 mL stainless steel jar and charged with one stainless steel ball (10 mm, 4.1 g). The reaction mixture was milled at 30 Hz for one hour. The residue was then transferred into a separating funnel, washing with dichloromethane (30 mL) and water (30 mL). The layers were separated and the aqueous layer further extracted with dichloromethane (2 x 30 mL). The aqueous phase was acidified to pH 1 with HCl (1 M) then extracted with dichloromethane (3 x 30 mL). This organic phase was dried ($MgSO_4$), filtered and the solvent removed under reduced pressure to yield benzoic acid (0.026 g, 0.21 mmol., 84%).

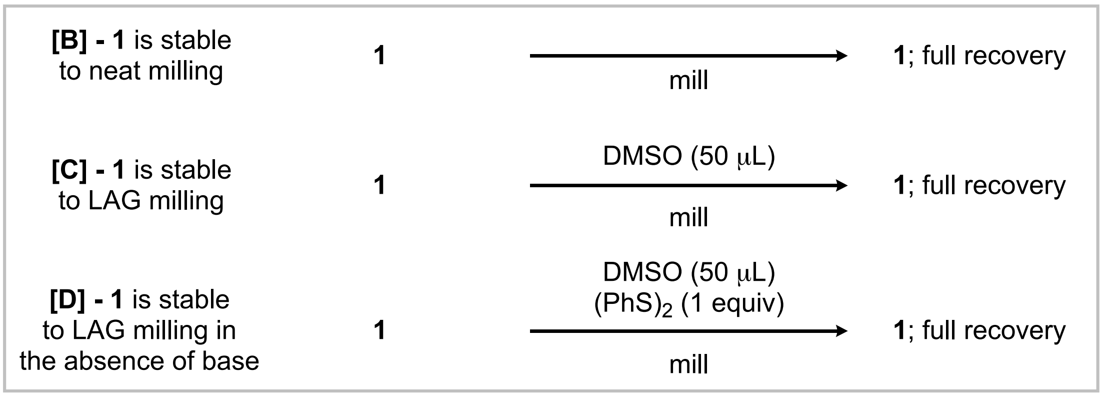

2,2-difluoro-1,3-diphenylpropane-1,3-dione **1** (0.065 g, 0.25 mmol.) and the specified additive(s) were added to a 14 mL stainless steel jar and charged with one stainless steel ball (10 mm, 4.1 g). The reaction mixture was milled at 30 Hz for one hour. The resulting mixture was transferred into a flask by manually removing the material with a spatula and washing with ethyl acetate (approximately 40 mL). The insoluble material was removed by gravity filtration and ^{19}F NMR was taken of the crude reaction mixture. In all cases the only peak observed was due to the starting material.

Testing intermediate

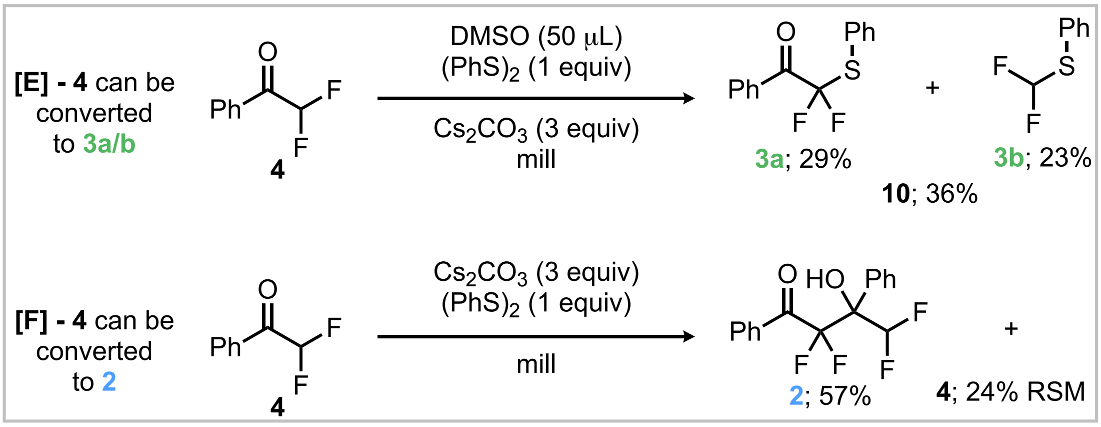

2,2-difluoro-1-phenylethan-1-one (0.039 g, 0.25 mmol.), cesium carbonate (0.244 g, 0.75 mmol), DMSO (0.050 mL, where applicable) and diphenyl disulfide (0.055 g, 0.25 mmol) were added to a 14 mL stainless steel jar and charged with one stainless steel ball (10 mm, 4.1 g). The reaction mixture was milled at 30 Hz for one hour. The resulting mixture was transferred into a flask by manually removing the material with a spatula and washing with ethyl acetate (approximately 40 mL). The insoluble material was removed by gravity filtration. α,α,α-trifluorotoluene (0.020 mL, 0.16 mmol) was added as a NMR standard and ^{19}F NMR was taken of the crude reaction mixture to determine the yields. To measure the quantity of benzoic acid (for the LAG case), the residue was then transferred into a separating funnel, washing with dichloromethane (30 mL) and water (30 mL). The layers were separated and the aqueous layer further extracted with dichloromethane (2 x 30 mL). The aqueous phase was acidified to pH 1 with HCl (1 M) then extracted with dichloromethane (3 x 30 mL). This organic phase was dried ($MgSO_4$), filtered and the solvent removed under reduced pressure to yield benzoic acid (0.011 g, 0.09 mmol., 36%).

Testing reversibility in solution

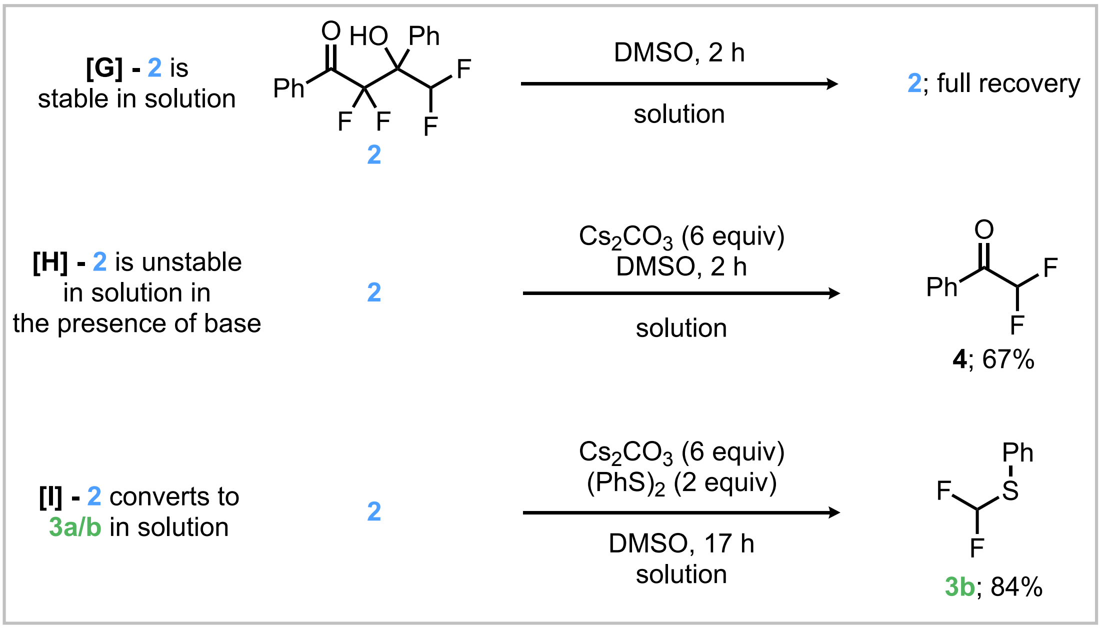

A mixture of 2,2,4,4-tetrafluoro-3-hydroxy-1,3-diphenylbutan-1-one (0.037 g, 0.12 mmol.), other reagents (see scheme above) in DMSO (1 mL) was stirred at room temperature for the required time. α,α,α-trifluorotoluene (0.020 mL, 0.16 mmol) was added as a NMR standard and ^{19}F NMR was taken of the crude reaction mixture to determine the yields.

Testing reversibility in ball mill

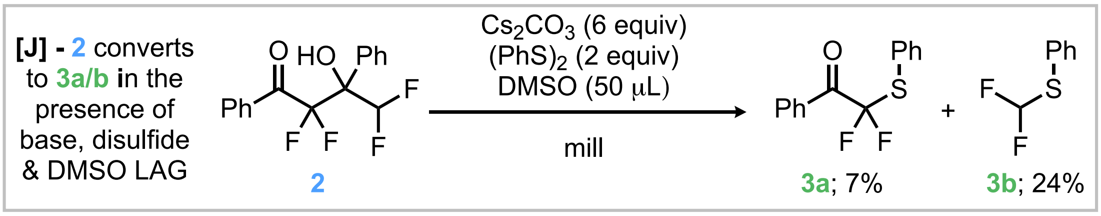

2,2,4,4-tetrafluoro-3-hydroxy-1,3-diphenylbutan-1-one (0.037 g, 0.12 mmol.), diphenyl disulfide (0.055 g, 0.25 mmol.), DMSO (0.050 mL) and cesium carbonate (0.244 g, 0.75 mmol.) were added to a 14 mL stainless steel jar and a ball added. The mixture was milled for one hour. The resulting mixture was transferred into a flask by manually removing the material with a spatula and washing with ethyl acetate (approximately 40 mL). The insoluble material was removed by gravity filtration. α,α,α-trifluorotoluene (0.020 mL, 0.16 mmol) was added as a NMR standard and ^{19}F NMR was taken of the crude reaction mixture to determine the yields.

Effect of time on yield for LAG reaction

The reactions were run as in GP1 but for different times.

Time / min	Yield 2	Yield 3a	Yield 4
10	22%	13%	35%
20	26%	27%	27%
30	12%	43%	20%
40	10%	53%	17%
50	0%	57%	8%
60	0%	62%	5%

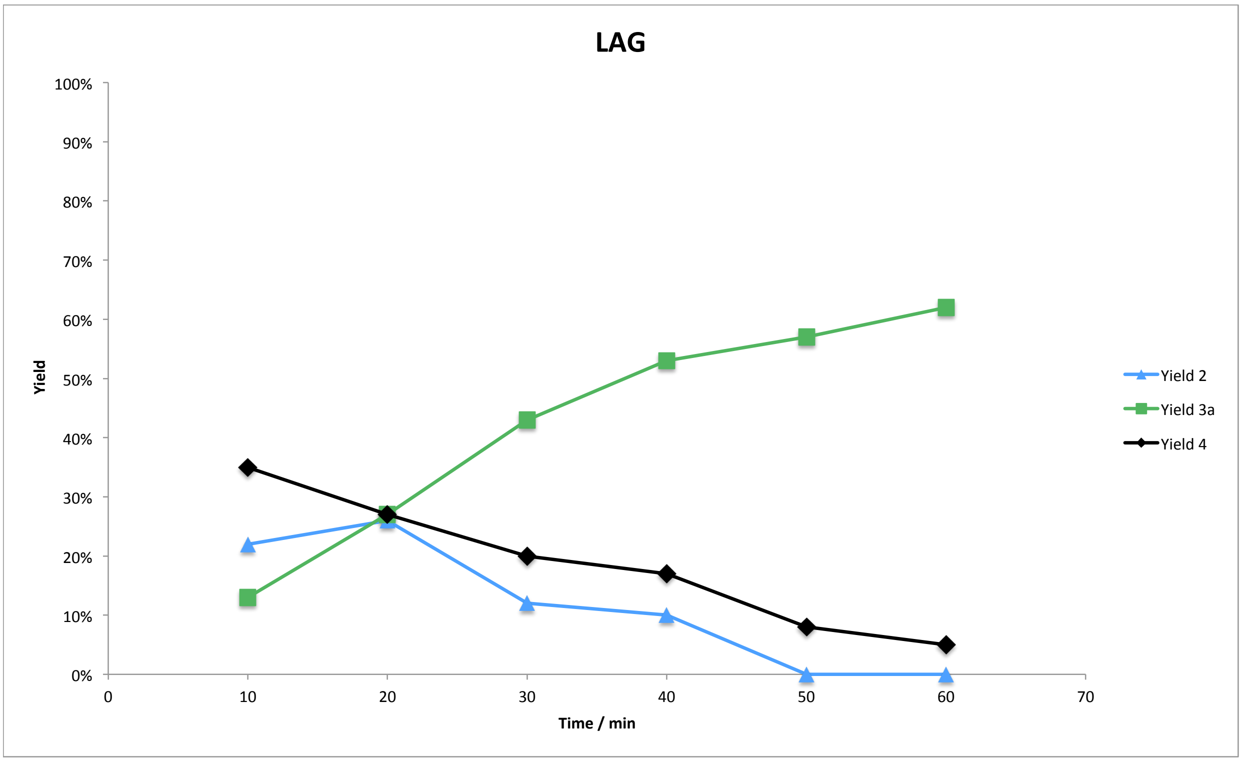

Effect of time on yield for neat grinding reaction

The reactions were run as in GP2 but for different times.

Time / hours	Yield 2	Yield 3a	Yield 4
1	88%	0%	10%
2	24%	10%	33%
4	24%	18%	21%
6	18%	26%	7%
8	14%	27%	8%

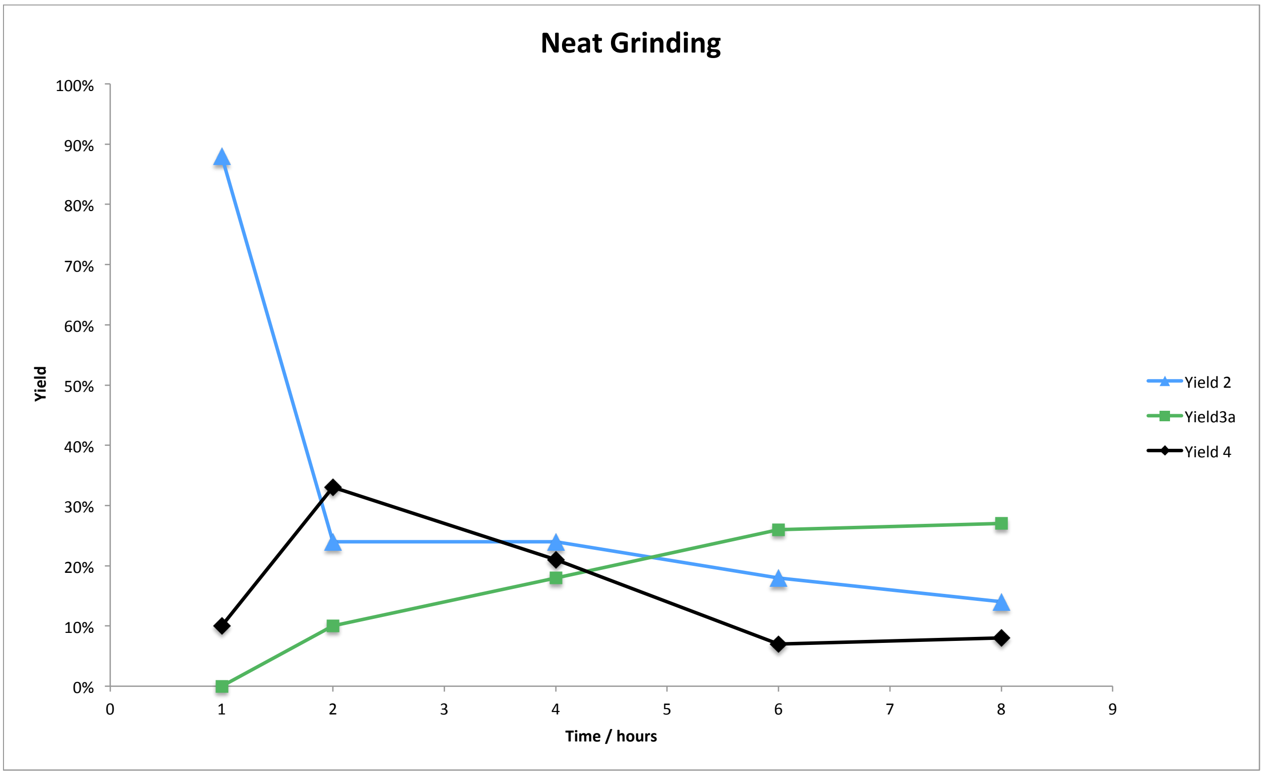

Effect of quantity of DMSO on LAG reaction

The reactions were run as in GP1 but for 10 minutes with different added quantities of DMSO.

DMSO added / μL	Yield 2	Yield 3a	Yield 4
0	50%	0%	29%
25	24%	5%	45%
50	22%	13%	35%
100	16%	48%	16%
150	12%	59%	9%

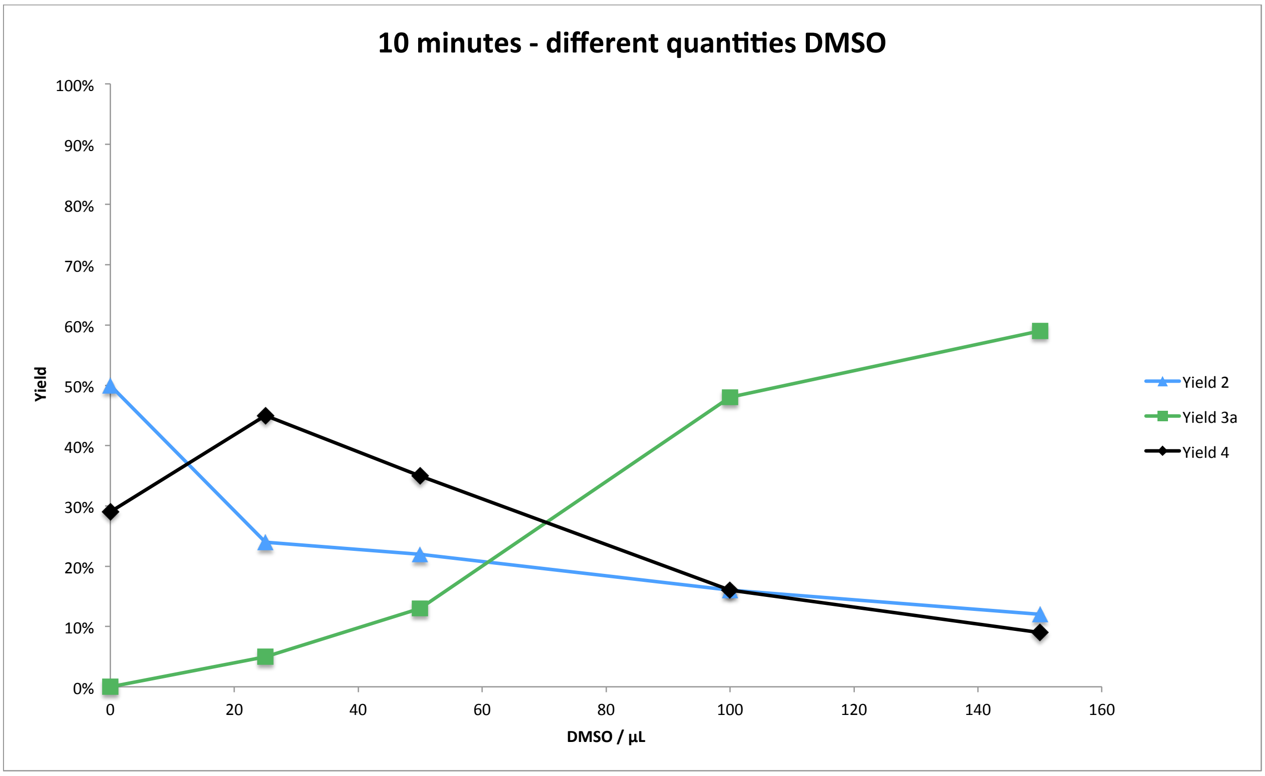

Solution comparison for different times

2,2-difluoro-1,3-diphenylpropane-1,3-dione (0.520 g, 2 mmol.), cesium carbonate (1.952 g, 6 mmol), diphenyl disulfide (0.440 g, 2 mmol) and DMSO (10 mL) were added to a flask, which was stirred at room temperature. α,α,α-trifluorotoluene (0.160 mL, 1.3 mmol) was added as a NMR standard. After stirring for the desired time, a sample was removed from the reaction mixture and filtered through cotton wool into an NMR tube. ^{19}F NMR was taken of this mixture and the yields determined.

Time / min	Yield 1	Yield 3a	Yield 4
3	68%	14%	18%
10	13%	33%	28%
20	0%	52%	28%
30	0%	62%	27%
40	0%	66%	27%
50	0%	68%	27%
60	0%	73%	27%

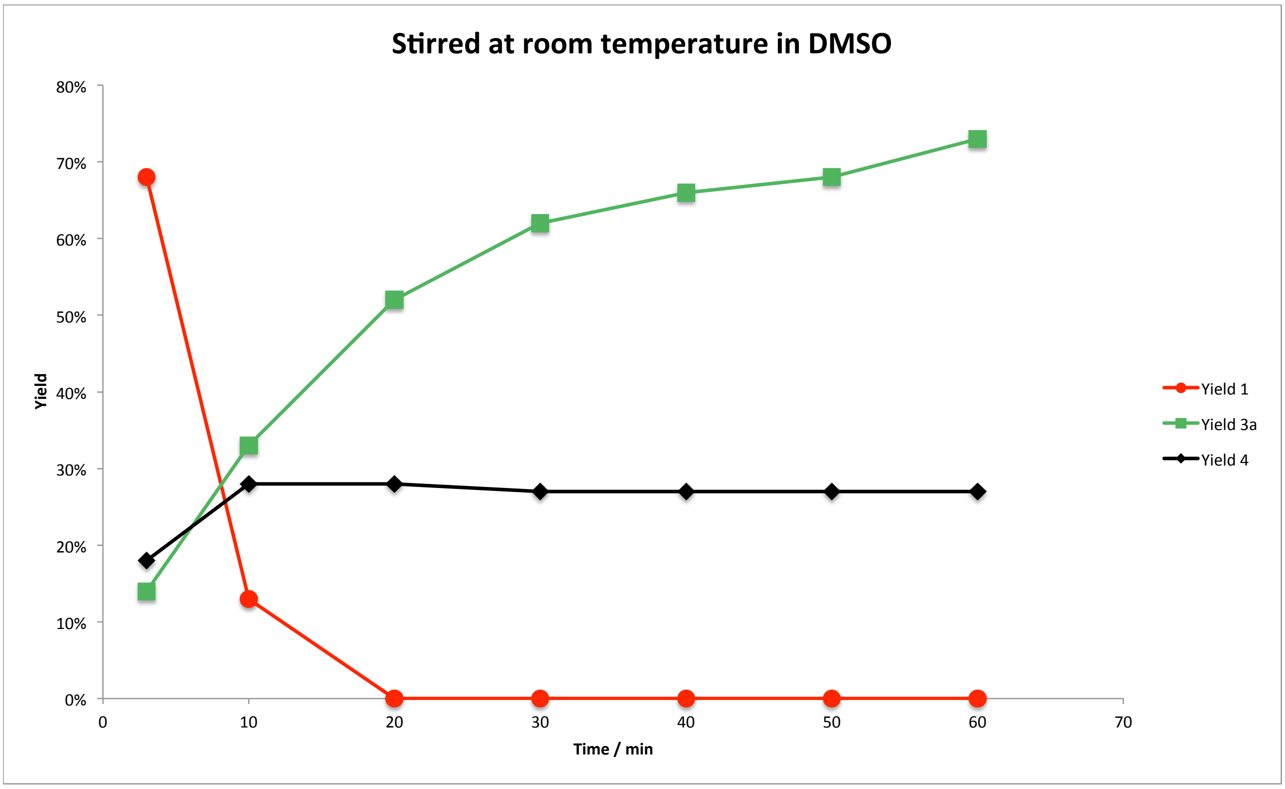

Synthesis of Starting materials

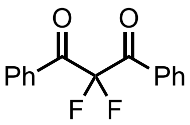

1

2,2-difluoro-1,3-diphenylpropane-1,3-dione (1)

To dibenzoylmethane (5.6 g, 25 mmol.), Selectfluor (17.7 g, 50 mmol.) and sodium carbonate (5.3 g, 50 mmol.) was added acetonitrile (250 mL). This mixture was stirred at room temperature for 72 h. The solvent was removed under reduced pressure, the residue dissolved in water (150 mL) and dichloromethane (150 mL) and transferred to a separating funnel. The layers were separated and the aqueous layer further extracted with dichloromethane (2 x 100 mL), the organic layers combined, dried ($MgSO_4$), filtered and the solvent removed under reduced pressure to yield the crude product. Hot hexane was added until most of the residue was dissolved, with a dark, insoluble impurity remaining. The solution in hot hexane was transferred to another flask and left to cool to room temperature. Colourless crystals of pure product were obtained and collected by vacuum filtration, washing on the filter with hexane to yield the desired product (4.69 g, 18 mmol., 72%).[2]

^{1}H NMR (400 MHz, $CDCl_3$) δ 8.09 (d, J = 8.5 Hz, 4H), 7.71 – 7.65 (m, 2H), 7.55 – 7.50 (m, 4H). **^{13}C NMR** (101 MHz, $CDCl_3$) δ 187.4 (t, J = 27.0 Hz), 135.1, 131.6 (t, J = 1.5 Hz),

130.3 (t, J = 2.5 Hz), 129.0, 112.7 (t, J = 266.0 Hz). **^{19}F NMR** (376 MHz, $CDCl_3$) δ -102.66 (s, 2F).

IR 1693, 1595, 1577, 1448, 1305, 1251, 1188, 1155, 1136, 1101, 999, 941, 887, 771, 719, 694, 678, 663, 569, 522, 435, 422 cm^{-1}

HRMS (EI+): [$C_{15}H_{10}F_2O_2$] [M+NH_4]$^+$ calc. 278.0987, found 278.0988.

mp 58 – 60 °C.

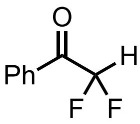

4

2,2-difluoro-1-phenylethan-1-one (4)

Prepared according to a modified literature procedure.[3] To ethylbenzoylacetate (4.3 mL, 4.77 g, 25 mmol.) was added aqueous sodium hydroxide (1 M, 25 mL) and the mixture stirred at room temperature for 20 hours. The mixture was transferred to a separating funnel and the aqueous layer washed with dichloromethane (3 x 10 mL). The aqueous layer was acidified to pH 1 with HCl (1 M). The precipitate was collected by vacuum filtration, washing on the filter with water to yield benzoylacetic acid (2.545 g, 15.5 mmol., 62%), which was used without further purification. To benzoylacetic acid (2.545 g, 15.5 mmol.) was added Selectfluor (13.718 g, 38.75 mmol.) and acetonitrile (100 mL). A reflux condenser was fitted and the mixture heated to 60 °C and stirred for 67 hours. The solvent was removed under reduced pressure. Water (50 mL) and dichloromethane (50 mL) were added to the residue and the mixture transferred to a separating funnel. The layers were separated and the aqueous layer further extracted with dichloromethane (2 x 50 mL), the organic layers combined, dried ($MgSO_4$), filtered and the solvent removed under reduced pressure to yield the crude product. This material was further purified by flash column chromatography with gradient elution (0 - 10% Ethyl acetate in petroleum ether) to yield the product as a colourless oil (1.146 g, 7.35 mmol., 47%).[3]

^{1}H NMR (500 MHz, $CDCl_3$) δ 8.08 (d, J = 7.5 Hz, 2H), 7.68 (t, J = 7.5 Hz, 1H), 7.54 (t, J = 7.9 Hz, 2H), 6.30 (t, J = 53.5 Hz, 1H). **^{13}C NMR** (126 MHz, $CDCl_3$) δ 187.6 (t, J = 25.5 Hz), 134.9, 131.5 (t, J = 2.0 Hz), 129.7 (t, J = 2.5 Hz), 129.0, 111.2 (t, J = 254.0 Hz). **^{19}F NMR** (471 MHz, CDCl3) δ -121.94 (d, J = 53.5 Hz, 2F).

IR 1707, 1597, 1579, 1450, 1348, 1288, 1055, 1001, 972, 933, 862, 761, 700, 682, 657, 572, 553, 468 cm^{-1}.

HRMS (EI+): [$C_8H_6F_2O$] calc. 156.0387, found 156.0385.

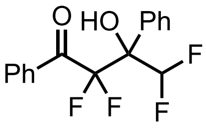

2

2,2,4,4-tetrafluoro-3-hydroxy-1,3-diphenylbutan-1-one (2)

2,2-difluoro-1,3-diphenylpropane-1,3-dione (0.065 g, 0.25 mmol.), diphenyl disulfide (0.055 g, 0.25 mmol.) and cesium carbonate (0.244 g, 0.75 mmol.) were added to a 14 mL stainless steel jar and a ball added. The mixture was milled for one hour. The residue was then transferred into a separating funnel, washing with ethyl acetate (30 mL) and water (30 mL). The layers were separated and the aqueous layer further extracted with ethyl acetate (2 x 30 mL). The combined organic phase was dried ($MgSO_4$), filtered and the solvent removed under reduced pressure. This reaction and workup procedure was repeated ten times and the crude material combined and purified by flash column chromatography with gradient elution (0 - 10% ethyl acetate in petroleum ether) to yield the product as a yellow oil (0.240 g, 0.77 mmol., 62%).

References

1 J. Phetcharawetch, N. M. Betterley, D. Soorukram, M. Pohmakotr, V. Reutrakul and C. Kuhakarn, *European J. Org. Chem.*, 2017, **2017**, 6840–6850.

2 J. L. Howard, Y. Sagatov, L. Repusseau, C. Schotten and D. L. Browne, *Green Chem.*, 2017, **19**, 2798–2802.

3 Y.-L. Li, J. Li and J. Deng, *Adv. Synth. Catal.*, 2017, **359**, 1407–1412.

Spectroscopic data

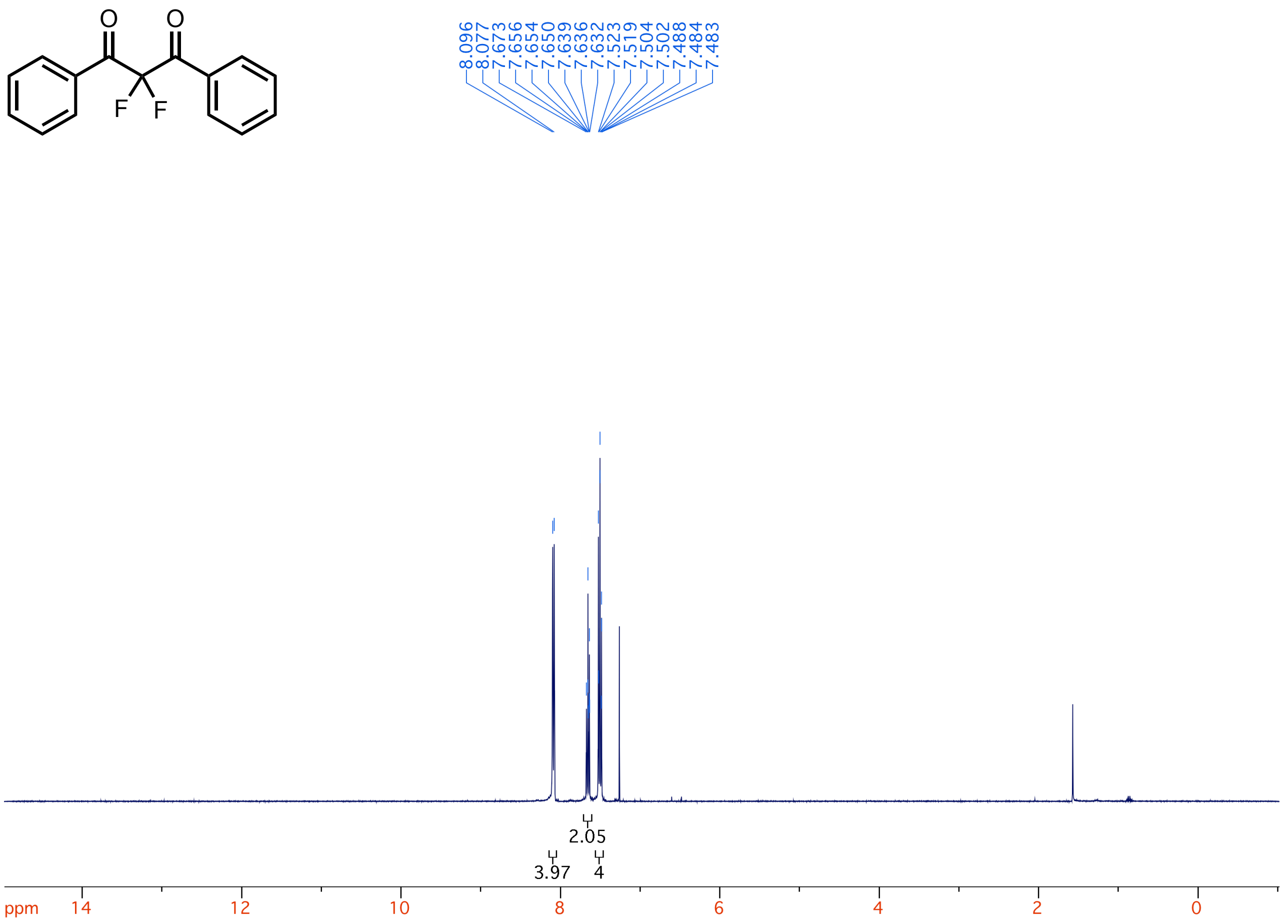

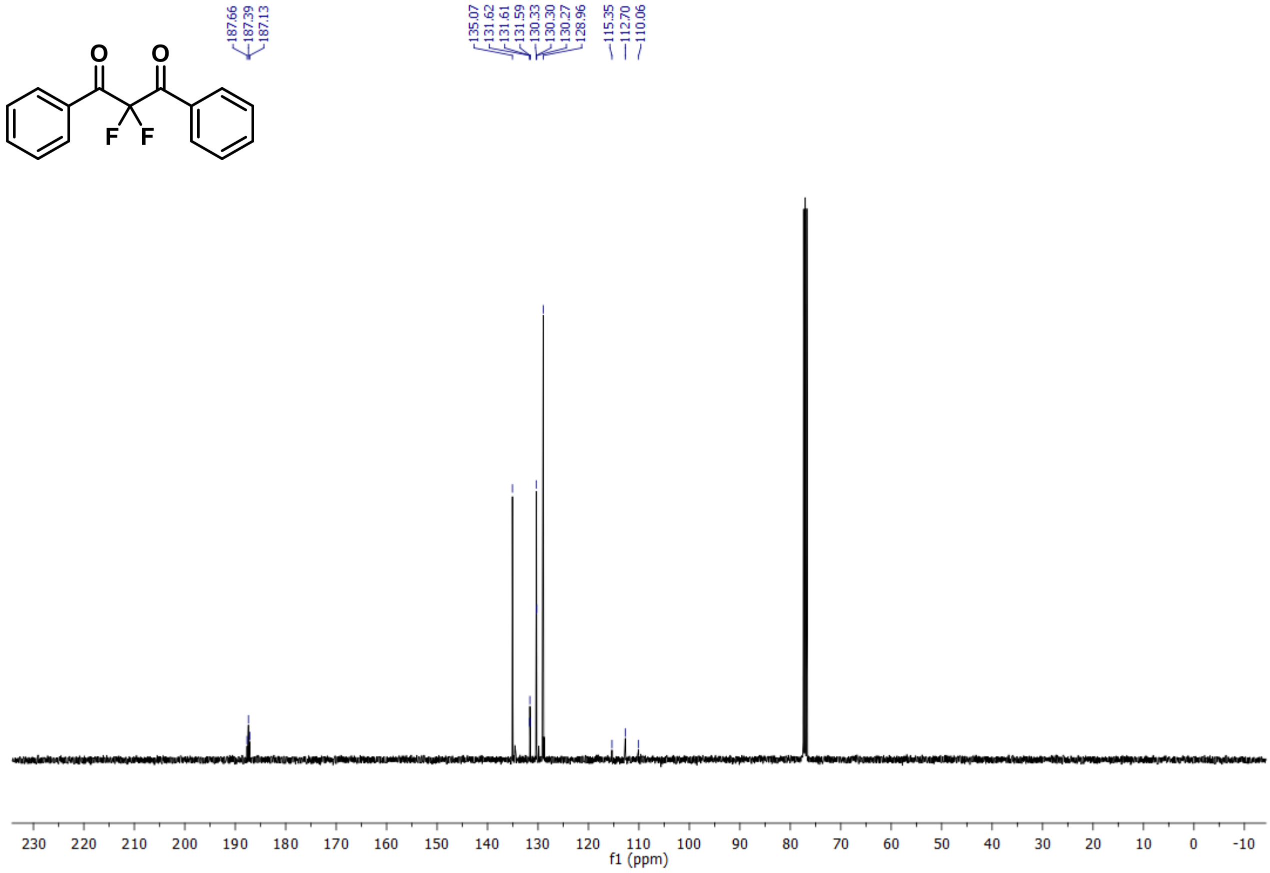

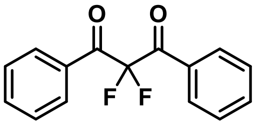
O
O
F
F

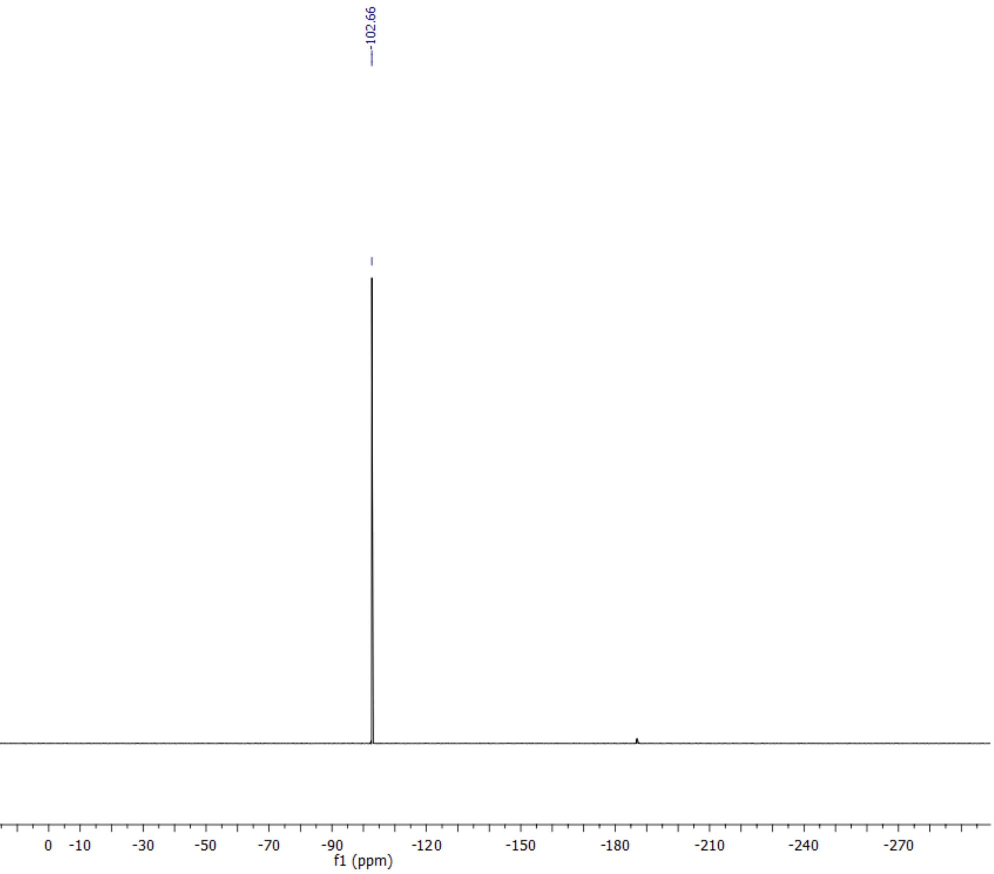
-102.66
80
60
40
20
0
-10
-30
-50
-70
-90
-120
-150
-180
-210
-240
-270
f1 (ppm)

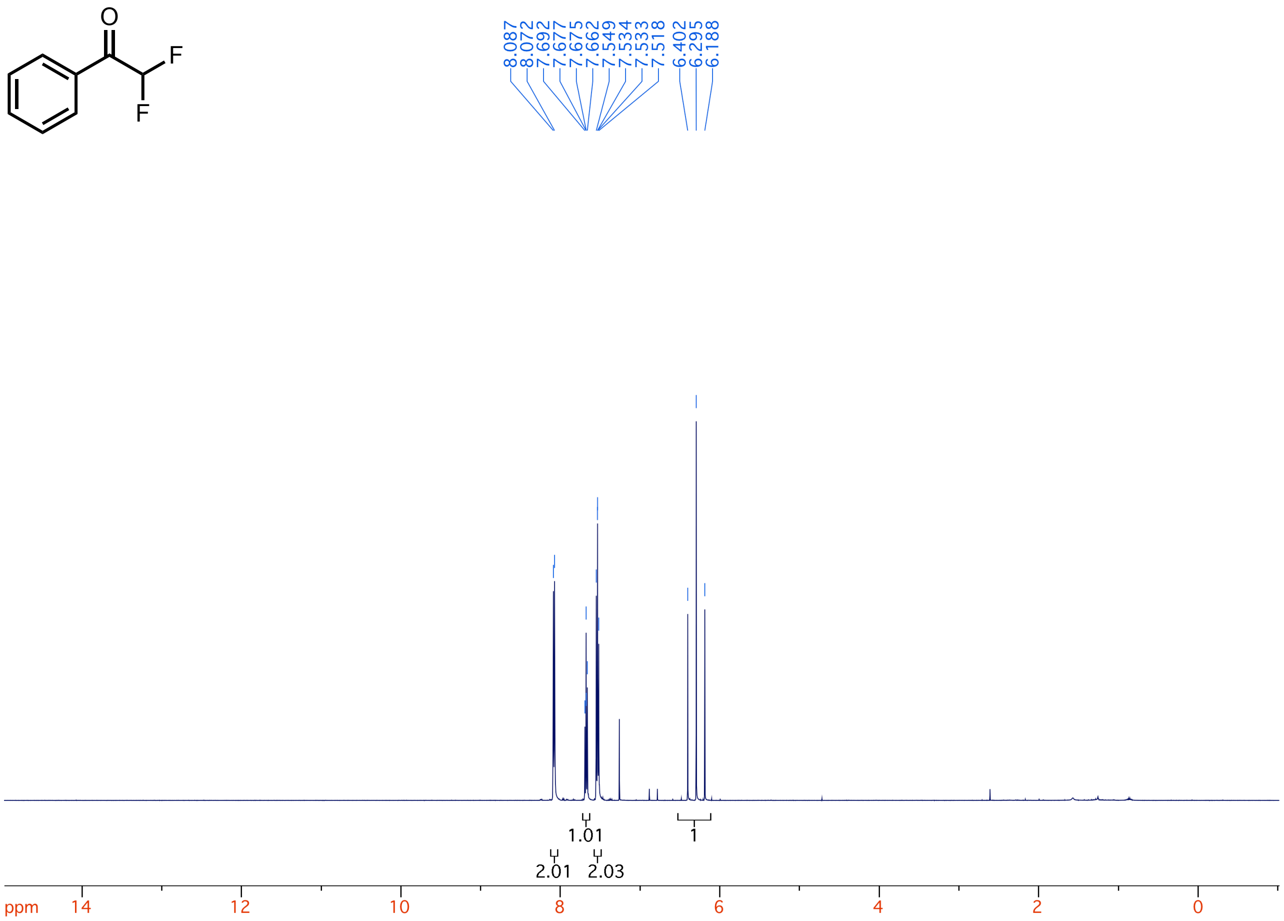
O
F
F
8.087
8.072
7.692
7.677
7.675
7.662
7.549
7.534
7.533
7.518
6.402
6.295
6.188
1.01
1
2.01
2.03
ppm
14
12
10
8
6
4
2
0

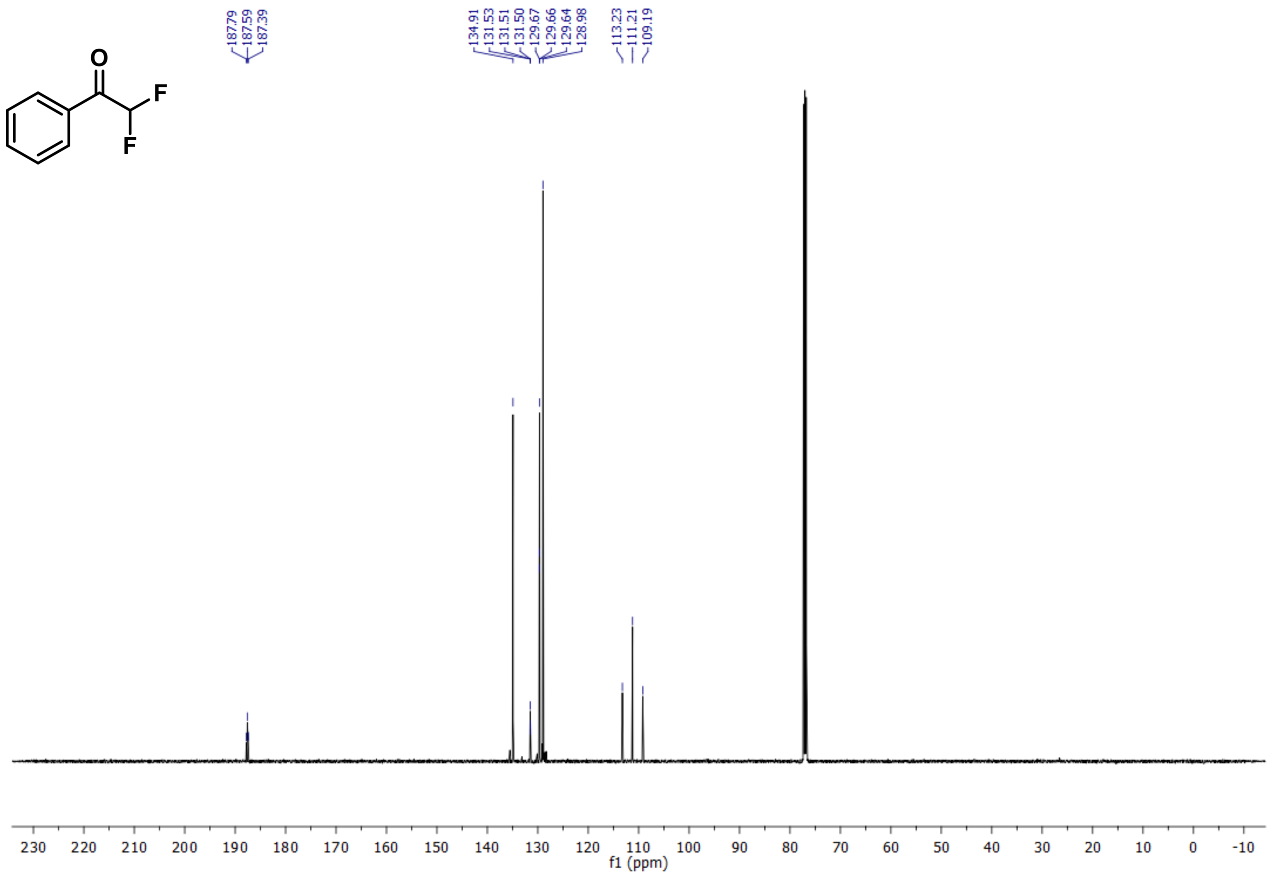
O
F
F
187.79
187.59
187.39
134.91
131.53
131.51
131.50
129.67
129.66
129.64
128.98
113.23
111.21
109.19
230 220 210 200 190 180 170 160 150 140 130 120 110 100 90 80 70 60 50 40 30 20 10 0 -10
f1 (ppm)

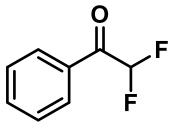

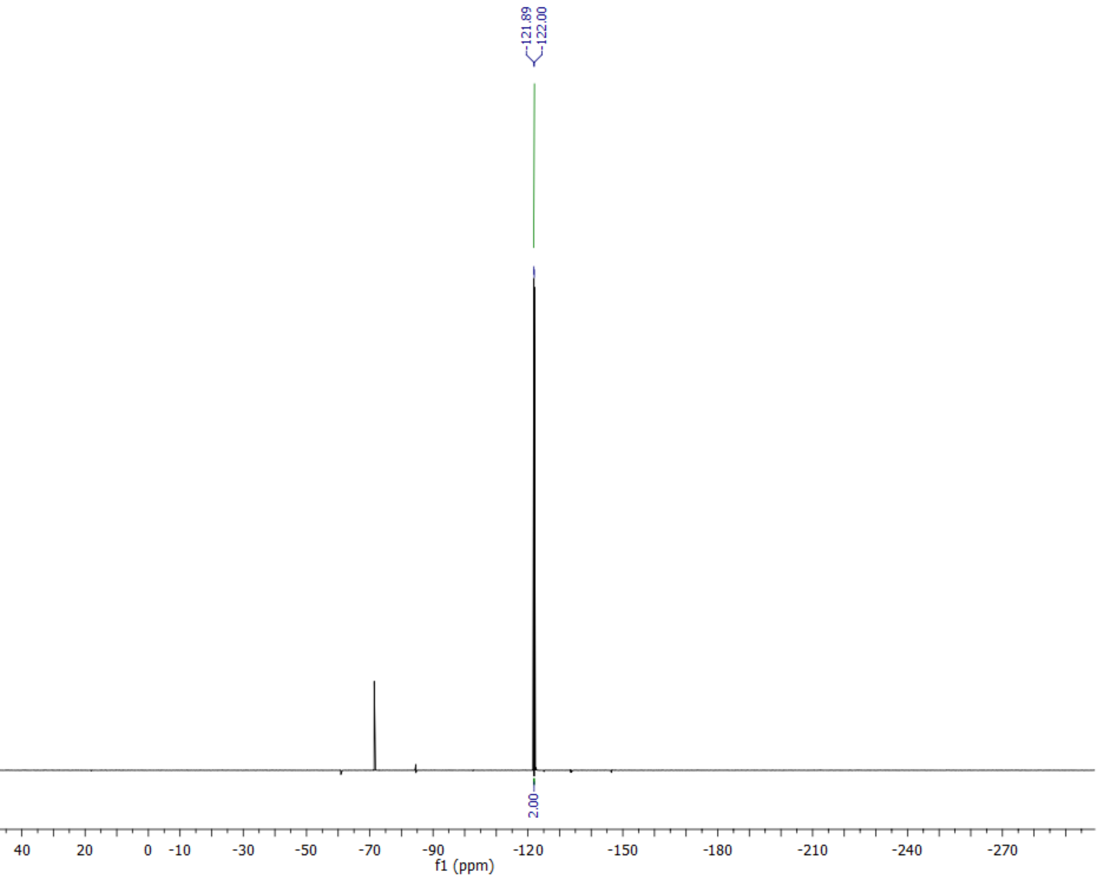

-121.89
-122.00
2.00
80
60
40
20
0
-10
-30
-50
-70
-90
-120
-150
-180
-210
-240
-270
f1 (ppm)

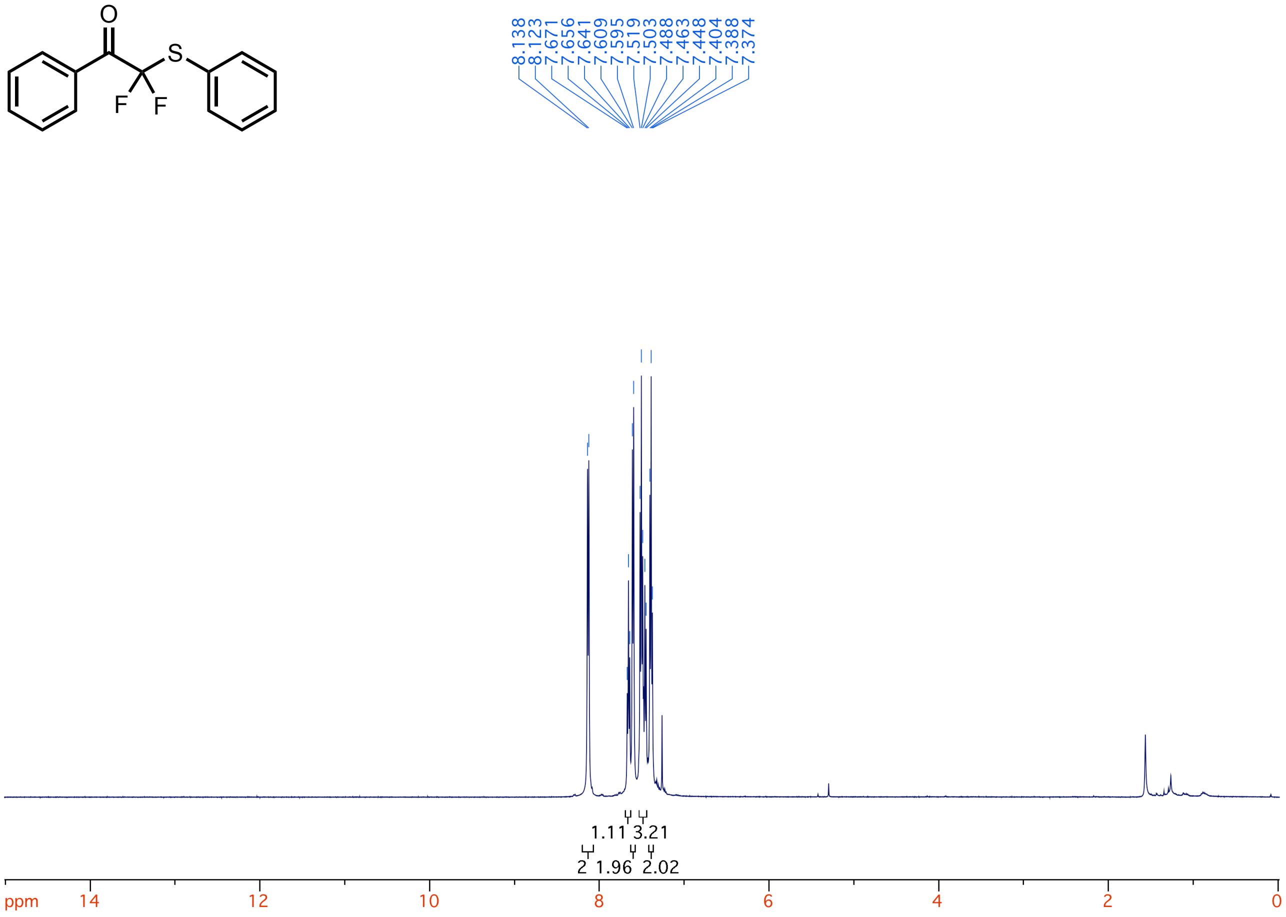
O
S
F F
8.138
8.123
7.671
7.656
7.641
7.609
7.595
7.519
7.503
7.488
7.463
7.448
7.404
7.388
7.374
1.11
3.21
2
1.96
2.02
ppm
14
12
10
8
6
4
2
0

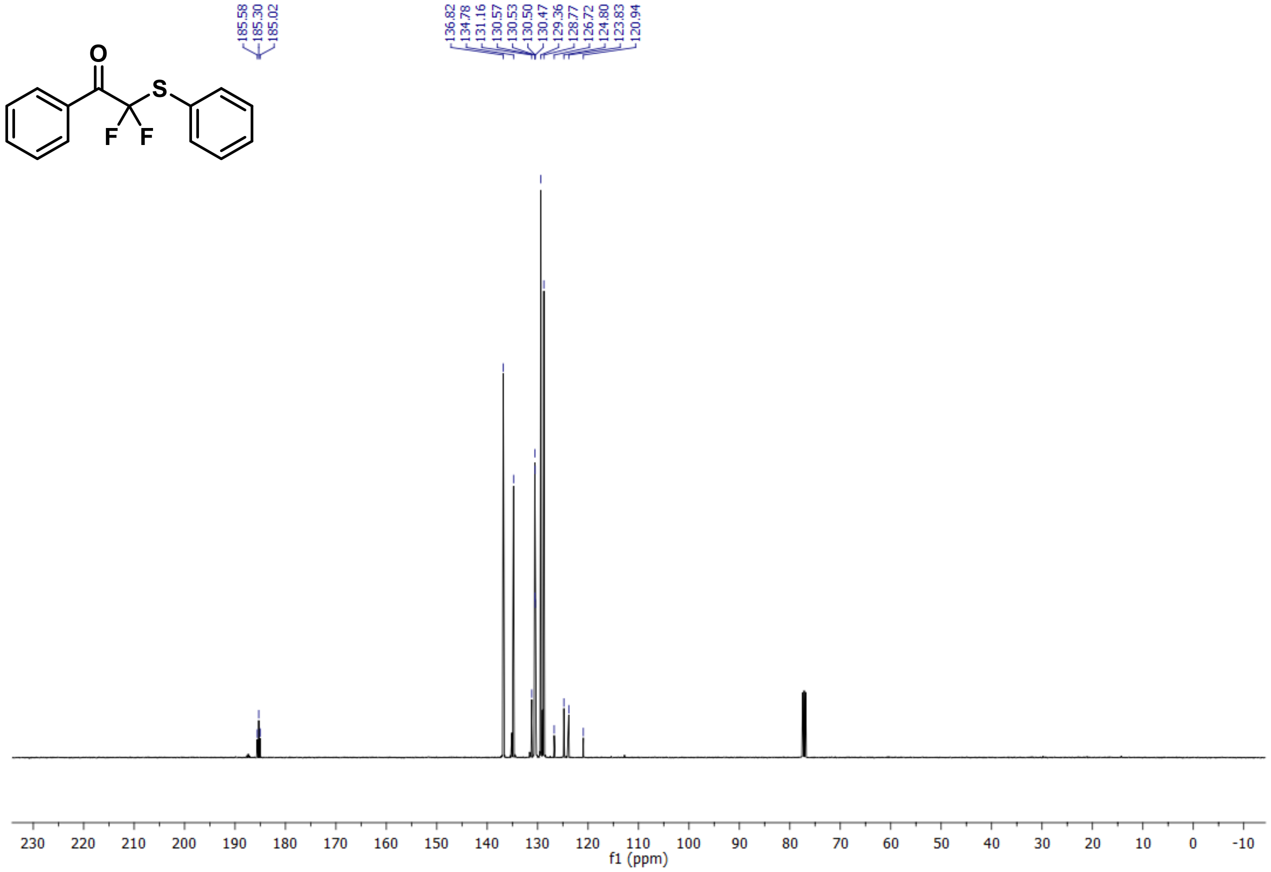
O
S
F F
185.58
185.30
185.02
136.82
134.78
131.16
130.57
130.53
130.50
130.47
129.36
128.77
126.72
124.80
123.83
120.94
230 220 210 200 190 180 170 160 150 140 130 120 110 100 90 80 70 60 50 40 30 20 10 0 -10
f1 (ppm)

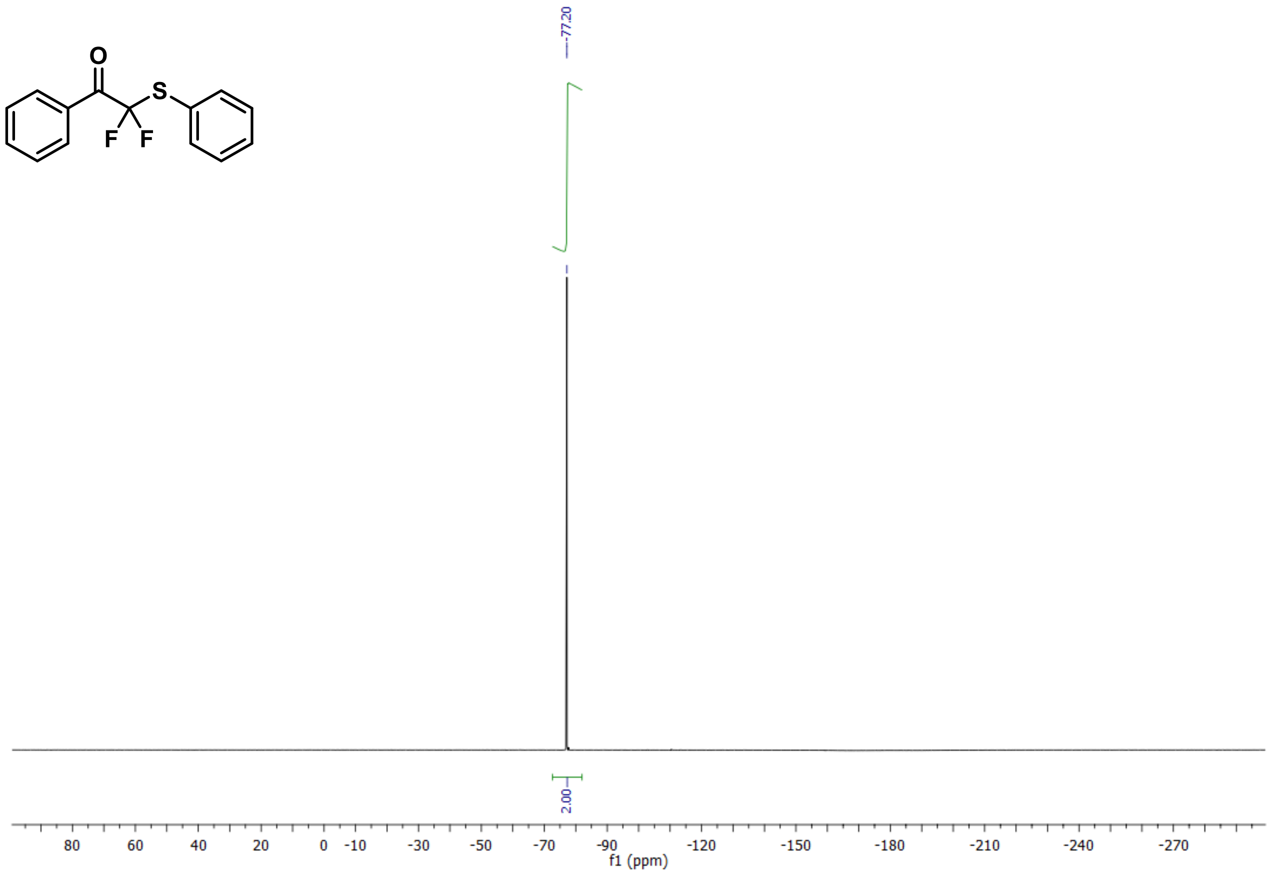
O
S
F
F
-77.20
2.00
80
60
40
20
0
-10
-30
-50
-70
-90
-120
-150
-180
-210
-240
-270
f1 (ppm)

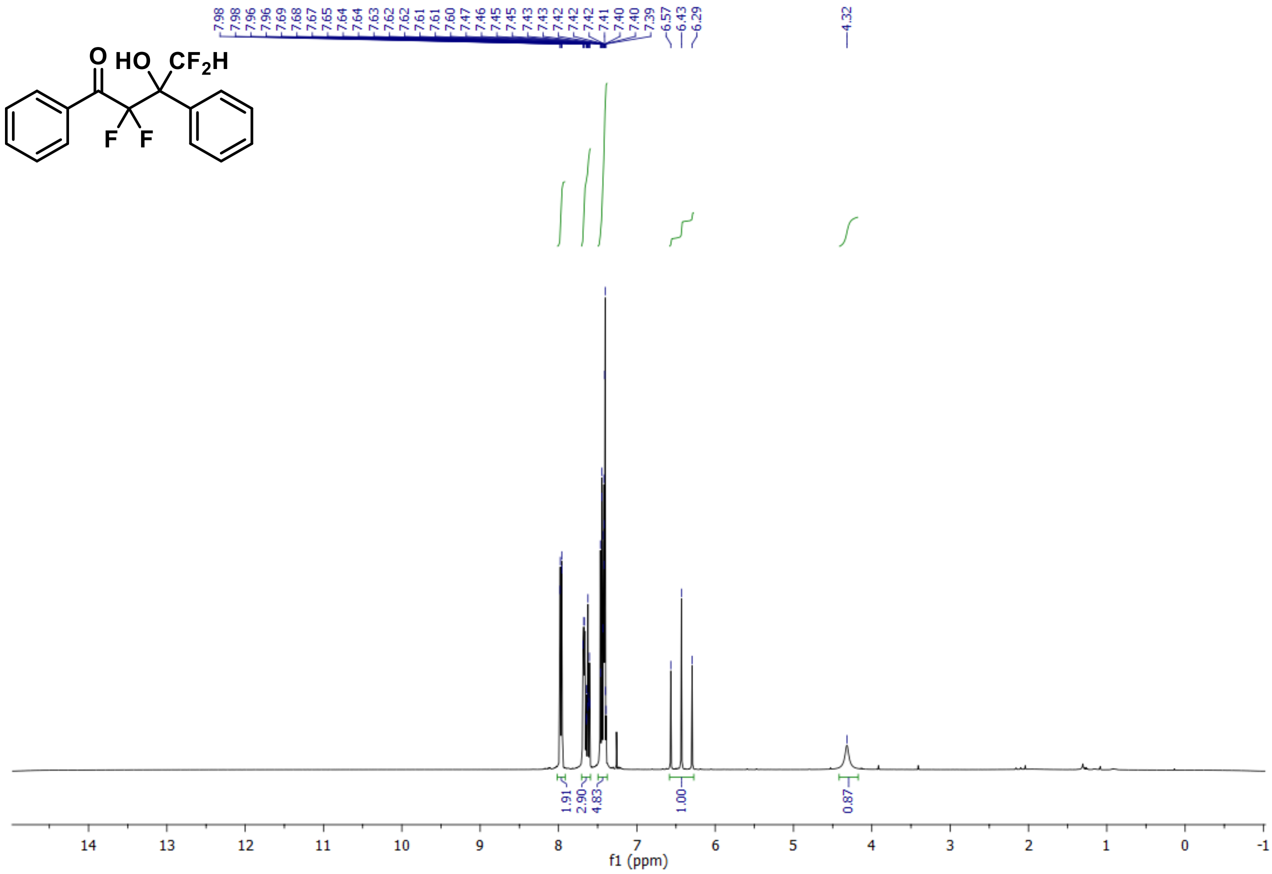
O HO CF2H
F F
7.98 7.98 7.96 7.96 7.69 7.68 7.67 7.65 7.64 7.64 7.63 7.62 7.62 7.61 7.61 7.60 7.47 7.46 7.45 7.45 7.43 7.43 7.42 7.42 7.42 7.41 7.40 7.40 7.39 6.57 6.43 6.29
4.32
1.91
2.90
4.83
1.00
0.87
14 13 12 11 10 9 8 7 6 5 4 3 2 1 0 -1
f1 (ppm)

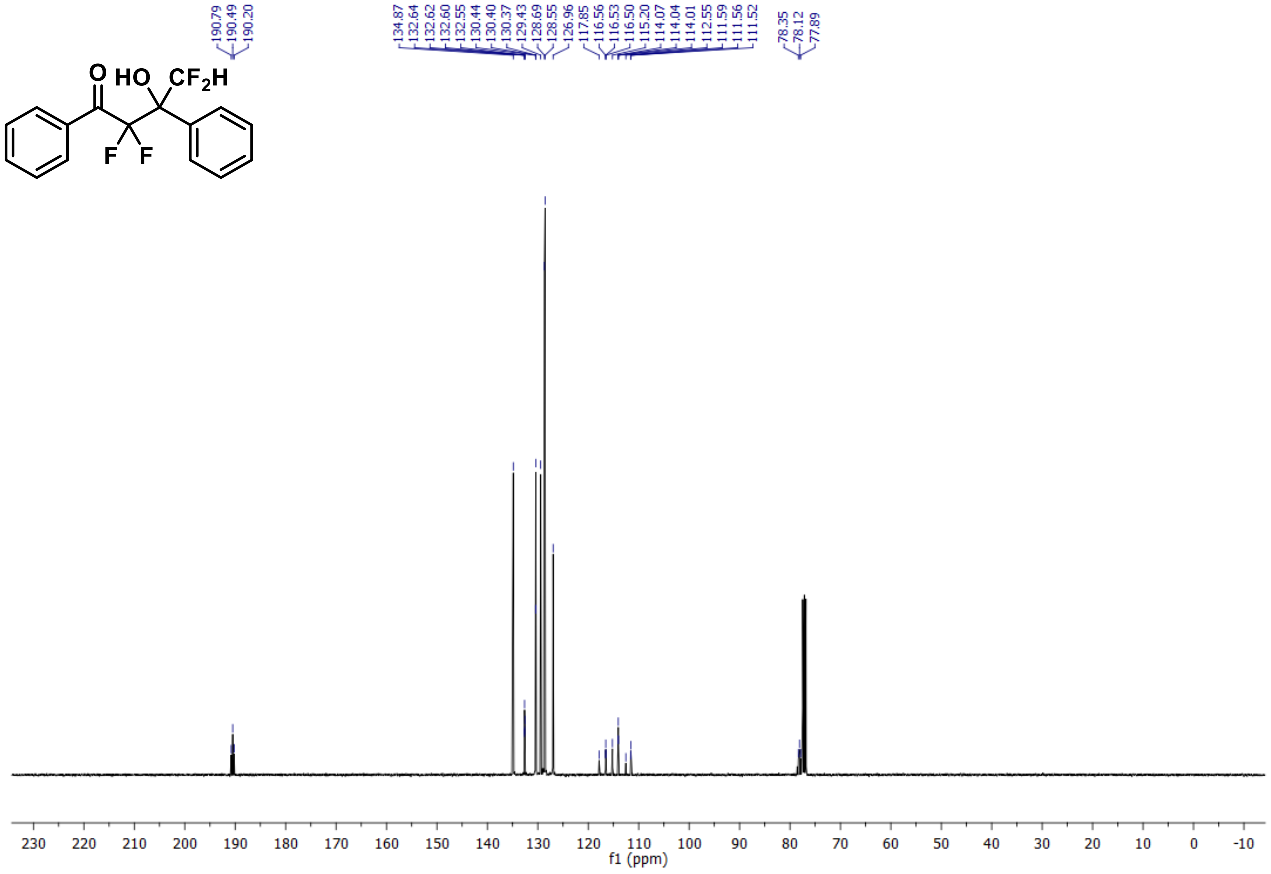
O HO CF2H
F F
190.79 190.49 190.20
134.87 132.64 132.62 132.60 132.55 130.44 130.40 130.37 129.43 128.69 128.55 126.96 117.85 116.56 116.53 116.50 115.20 114.07 114.04 114.01 112.55 111.59 111.56 111.52
78.35 78.12 77.89
230 220 210 200 190 180 170 160 150 140 130 120 110 100 90 80 70 60 50 40 30 20 10 0 -10
f1 (ppm)

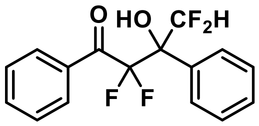

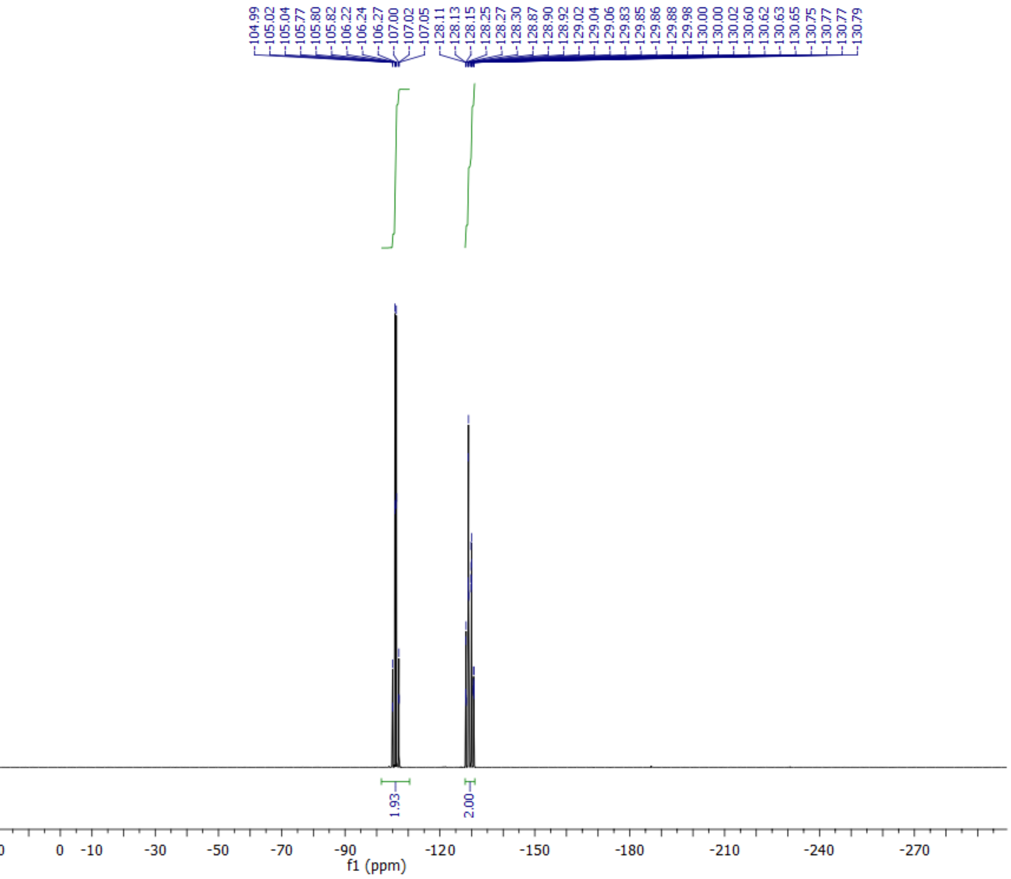
-104.99
-105.02
-105.04
-105.77
-105.80
-105.82
-106.22
-106.24
-106.27
-107.00
-107.02
-107.05
-128.11
-128.13
-128.15
-128.25
-128.27
-128.30
-128.87
-128.90
-128.92
-129.02
-129.04
-129.06
-129.83
-129.85
-129.86
-129.88
-129.98
-130.00
-130.02
-130.60
-130.62
-130.63
-130.65
-130.75
-130.77
-130.79
1.93
2.00
80
60
40
20
0
-10
-30
-50
-70
-90
-120
-150
-180
-210
-240
-270
f1 (ppm)
